# Energy substrate supplementation increases ATP levels and is protective to PD neurons

**DOI:** 10.1016/j.crphar.2024.100187

**Published:** 2024-05-23

**Authors:** Andrey Y. Vinokurov, Marina Y. Pogonyalova, Larisa Andreeva, Andrey Y. Abramov, Plamena R. Angelova

**Affiliations:** a Cell Physiology and Pathology Laboratory, Orel State University, Orel, Russia; b Mitocholine Ltd., London, UK; c Department of Clinical and Movement Neurosciences, UCL Queen Square Institute of Neurology, Queen Square, WC1N 3BG, London, UK

**Keywords:** Brain energy metabolism, Substrate supplementation, Neurodegeneration, Ageing, Parkinson's disease

## Abstract

Alteration of mitochondrial metabolism by various mutations or toxins leads to various neurological conditions. Age-related changes in energy metabolism could also play the role of a trigger for neurodegenerative disorders. Nonetheless, it is not clear if restoration of ATP production or supplementation of brain cells with substrates for energy production could be neuroprotective. Using primary neurons and astrocytes, and neurons with familial forms of neurodegenerative disorders we studied whether various substrates of energy metabolism could improve mitochondrial metabolism and stimulate ATP production, and whether increased ATP levels could protect cells against glutamate excitotoxicity and neurodegeneration. We found that supplementation of neurons with several substrates, or combination thereof, for the TCA cycle and cellular respiration, and oxidative phosphorylation resulted in an increase in mitochondrial NADH level and in mitochondrial membrane potential and led to an increased level of ATP in neurons and astrocytes. Subsequently, these cells were protected against energy deprivation during ischemia or glutamate excitotoxicity. Provision of substrates for energy metabolism to cells with familial forms of Parkinson's disease also prevented triggering of cell death. Thus, restoration of energy metabolism and increase of ATP production can play neuroprotective role in neurodegeneration. A combination of a succinate salt of choline and nicotinamide provided the best results.

## Highlights

• Mitochondrial substrates and combinations thereof optimises mitochondrial functionof primary neurons and astrocytes.
• DISU + NAM increases energy capacity of neurons and astrocytes.
• Supplementation of neurons and astrocytes with substrate combinations of DISU + NAM stimulates their ATP production.
• DISU + NAM protects iPSC-derived neurons and fibroblasts from patients with Parkinson's disease against cell death.

## Graphical abstract

**Figure:**
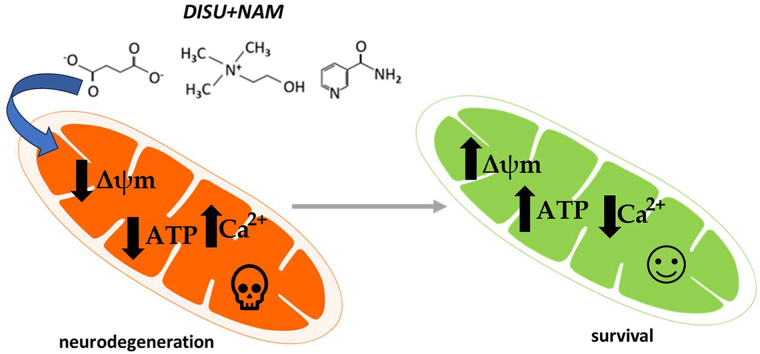

## 1 Introduction

Excitability of neurons requires large amounts of energy in comparison to other tissues and renders these types of cells heavily dependent on the ATP production rates. Most of the energy available for brain functions comes from glucose metabolism through the processes of glycolysis and oxidative phosphorylation in the mitochondria to yield ATP.

Although part of the ATP in the brain should be produced in glycolysis, mostly in glial cells but also in neurons as a part of the glucose metabolism, brain cells produce most of their energy via very effective process of oxidative phosphorylation to maintain ATP in conditions of high energy demand (Andrey et al., 2019). Bioenergetic activity is not identical across the brain and varies widely in different brain regions depending on the ATP demand (Cheng et al., 2021). Any change in mitochondrial bioenergetics reflects neuronal function and vice versa. Thus, almost all neurodegenerative diseases, including the most prevalent - Alzheimer's disease, Parkinson's disease and ALS, are characterized by mitochondrial dysfunction (Angelova et al., 2017; Joshi et al., 2018; Ludtmann et al., 2017) that leads to loss of neurons in specific brain regions. Mitochondrial mutations also induce alteration of energy metabolism in brain cells and lead to MELAS (mitochondrial myopathy, encephalopathy, lactic acidosis, and stroke-like episodes) and MERRF (myoclonic epilepsy and ragged-red fiber syndrome) that could also be induced by lack of ATP (Goto et al., 1990; Abramov et al., 2019). Ischemic conditions not only limit ATP production but can also initiate massive ATP consumption for the maintenance of ion gradients in the cells, additionally, it may be associated with glutamate excitotoxicity that has also been shown to be a highly energy expending process (Abramov et al., 2010; Nicholls et al., 2007). ATP deprivation could act as a trigger for seizures, but epileptic activity also leads to a massive ATP consumption (Kovac et al., 2012; Lombroso, 1996).

However, all these pathological conditions are not only associated with the lack of ATP but also with mitochondrially induced oxidative stress (Angelova, 2021; Angelova et al., 2016) and abnormal mitophagy (Pickrell et al., 2015). Mitochondrial dysfunction could then directly trigger apoptotic and necrotic cell death by the induction of the permeability transition pore (PTP) opening mediated by the overproduction of ROS and calcium homeostasis dysregulation (Rasola et al., 2011).

Considering this, it is not clear whether all forms of mitochondrial dysfunction evoked by ATP depletion, or other than ATP-related factors, lead to pathology in neurons. In order to test if an increase in ATP levels can be neuroprotective in pathological conditions, we used various mitochondrial substrates for the activation of bioenergetics and stimulation of ATP production. In addition, we used these ATP-increasing compounds to protect neurons and patients' fibroblasts with familial form of Parkinson's disease against cell death. We also used a model of glutamate excitotoxicity to test if elevation of ATP level in primary neurons can be protective.

We have found that the mitochondrial substrates pyruvate, succinate, choline, a succinate salt of choline and nicotinamide - and combinations of these compounds increased mitochondrial membrane potential, NADH pool in mitochondria, and boost ATP levels. The combination of succinate salt of choline and nicotinamide was the most effective. The compounds that most effectively increased ATP levels were the ones that further protected cells with familial forms of Parkinson's disease in a cell death assay and neurons against glutamate excitotoxicity.

## 2 Materials and methods

All standard laboratory chemicals were purchased from Sigma Aldrich (Gillingham, UK) and Fisher Scientific (Loughborough, UK) unless otherwise stated. Choline succinate salt 2:1 (DISU) and a composition of DISU and nicotinamide (NAM) at molar ratio 1:0.4 were provided by Mitocholine Ltd. (London, UK).

### 2.1 Cell cultures

#### 2.1.1 Primary cell culture preparation

Mixed neuronal brain cultures were prepared from Sprague-Dawley rat pups 0–3 days postpartum (UCL breeding colony). Animal husbandry and experimental procedures were performed in full compliance with the United Kingdom Animal (Scientific Procedures) Act of 1986. Subjects were culled using a Schedule 1 procedure and the brain was dissected into ice-cold PBS solution (Ca2+, Mg2+-free; Gibco-Invitrogen, Paisley, UK). The tissue was minced and trypsinized (0.25% for 15 min at 37 °C), triturated and plated on poly-D-lysine-coated 25 mm glass coverslips and cultured in Neurobasal-A medium (Gibco-Invitrogen) supplemented with B-27 (Gibco-Invitrogen) and 2 mM Glutamax using routine protocol (Angelova et al., 2023). Cultures were maintained at 37 °C in a humidified atmosphere of 5% CO2, fed once a week and maintained for a minimum of 14 days before experimental use. Neurons were easily distinguishable from glia: they appeared bright using phase contrast, had smooth rounded somata and distinct processes, and lay just above the focal plane of the glial layer. Cells were used at 14–16 days in vitro (DIV).

#### 2.1.2 Human skin fibroblasts culture

Fibroblasts were generated from a 4-mm skin punch biopsy taken under local anesthetic following local ethical approval and full informed consent. Human skin fibroblasts were cultured in DMEM media, supplemented with 10% fetal bovine serum (Sigma-Aldrich) at 37 °C, 5% CO2, and 95% humidity.

#### 2.1.3 Neuronal differentiation of NPC

Neural progenitor cells were dissociated from cell clusters using Accutase (Sigma-Aldrich) and seeded onto low-adhesion 6-well plates in NPC media containing DMEM/F12, N2 (Thermo Fisher Scientific), B27 (Thermo Fisher Scientific), Pen/Strep (Thermo Fisher Scientific) and FGF2 (Thermo Fisher Scientific). For differentiation, the medium containing Neurobasal (Thermo Fisher Scientific), Pen/Strep (Thermo Fisher Scientific), B27 (Thermo Fisher Scientific), SU5402 (Sigma-Aldrich), PD0325901 (Sigma-Aldrich), and DAPT (Sigma-Aldrich) (“differentiation medium”) was added and kept for 2 days. The differentiation medium was renewed every day. On the third day, cells were detached incubation in Accutase at 37 °C, washed and plated onto poly-L-lysine/laminin/fibronectin-coated (Sigma-Aldrich) 8-well ibidi plates in neuronal maturation medium supplemented with ROCK inhibitor Y27632 (Selleckchem) for 24 h. Neuronal maturation medium was composed of Neurobasal (Thermo Fisher Scientific) supplemented with B27, glutamine, Pen/Strep, BDNF and GDNF (Peprotech), ascorbic acid (Sigma-Aldrich), Laminin (Sigma-Aldrich), DAPT (Sigma-Aldrich), and dbcAMP (Selleckchem). On the next day the ROCK inhibitor was removed, and half of the medium was replaced with fresh neuronal maturation medium three times a week.

### 2.2 Live cell imaging

Live cell imaging experiments were performed using a Zeiss LSM 710 visible confocal laser scanning microscopy (VIS CLSM) equipped with a META detection system or Zeiss 900 CLSM (Carl Zeiss Microscopy GmbH, Jena, Germany) and a 40 × oil immersion objective, using Zen Blue software (Zeiss), unless otherwise stated.

#### 2.2.1 Mitochondrial membrane potential

The mitochondrial membrane potential (Δψm) was measured by loading cells with 25 nM tetramethyl rhodamine methyl ester (TMRM) (Invitrogen by Thermo Fisher Scientific, USA) in a HEPES-buffered HBSS for 40 min at room temperature. Measurements were obtained by using × 63 oil immersion objective whilst keeping 25 nM TMRM in the imaging solution. TMRM was excited using the 561 nm laser line and fluorescence was measured above 580 nm. Z-stack images were obtained by confocal microscopy and the basal Δψm was measured. TMRM was used in the redistribution mode to assess the Δψm, and therefore a reduction in TMRM fluorescence represents mitochondrial depolarization.

#### 2.2.2 NADH measurements

NADH autofluorescence was measured using an epifluorescence inverted microscope equipped with a × 40 fluorite objective. Excitation light at a wavelength of 360 nm was provided by a Xenon arc lamp, the beam passing through a monochromator (Cairn Research, Faversham, Kent, UK). Emitted fluorescence light was reflected through a 455 nm long-pass filter to a cooled CCD camera (Retiga, QImaging) and digitized to 12-bit resolution. Imaging data were collected and analyzed using Andor iQ3 software (Belfast, UK).

#### 2.2.3 ATP levels assay

To determine the ATP levels, primary neuroglial co-cultures were transfected with a mitochondrially-targeted genetically-encoded ATP probe (AT1.03), using Effectene according to the manufacturer's instructions (Qiagen). The FRET signal was quantified by the 527:475 nm ratio following an excitation of 405 nm and a filter from 515 to 580 nm (Sokolovski et al., 2021).

#### 2.2.4 Metabolic state assay

To assess the ATP levels (which correlate to cytosolic Mg2+ changes), ATP consumption rate and time to bioenergetic collapse, [Mg2+]c was imaged using MagFura-2 AM (Molecular Probes). Fluorescence images were acquired at 20 s intervals on an epifluorescence inverted microscope equipped with a 20x fluorite objective (excitation at 340 and 380 nm). The emitted light was reflected through a 515 nm long-pass filter to a cooled CCD camera (Retiga; QImaging) and digitized to 12-bit resolution (Cairn Research, UK). Andor iQ3 was employed for data collection and analysis.

#### 2.2.5 Glutamate excitotoxicity

Hippocampal or cortical neurons were loaded for 30 min at room temperature with 5 μM Fura-FF AM (Molecular Probes) and 0.005% Pluronic acid in an HBSS: 156 NaCl, 3 KCl, 2 MgSO4, 1.25 KH2PO4, 2 CaCl2, 10 glucose, and 10 HEPES, pH adjusted to 7.35. For simultaneous measurement of [Ca2+]c and mitochondrial membrane potential (Δψm), Rh123 (1 μM, Thermo Fisher Scientific) was added into the cultures during the last 15 min of the Fura-FF loading period, and the cells were then washed three to five times before an experiment. Fluorescence measurements were obtained on an epifluorescence inverted microscope equipped with a 20 × fluorite objective. [Ca2+]c and Δψm were monitored in single cells using excitation light provided by a xenon arc lamp, with the beam passing sequentially through 10 nm bandpass filters centered at 340, 380, and 490 nm housed in a computer-controlled filter wheel (Cairn Research). Emitted fluorescence light was reflected through a 515 nm long-pass filter to a cooled CCD camera (Retiga, QImaging). The Fura-FF data have not been calibrated in terms of [Ca2+]c because of the uncertainty arising from the use of different calibration techniques. For Δψm measurements, we have normalized the fluorescence signals between resting level (set to 0%) and a maximal signal (which correspond to full mitochondrial depolarization) generated in response to the protonophore FCCP (carbonyl cyanide-p-trifluoromethoxyphenylhydrazone; 1 μM; set to 100%). Changes in Rh123 fluorescence were then expressed as the difference between peak values (at 15 min) attained during glutamate stimulation and basal values. All imaging data were collected and analyzed using iQ3 software from Andor.

#### 2.2.6 Cell death

For assessment of cell death in vitro, before imaging, cells were incubated with propidium iodide (PI; 10 μM) and 300 nM Hoechst for 15 min, washed 3 times with PBS 1 × and assayed using a cooled charge-coupled device (CCD) camera. Hoechst labels the total number of nuclei, whereas PI stains only cells with a disrupted plasma membrane. Dead cells (PI positive) were counted as a fraction of the total (Hoechst positive). In each experiment, 10 random fields were examined. The mean is representative of 3 independent experiments each for a separate culturing condition.

### 2.3 Statistical analysis

Statistical analysis (unpaired two sample *t*-test, or one-way analysis of variance (ANOVA), p value set at 0.05) and curve fitting were performed using Origin 2021 (Microcal Software Inc., Northampton, MA) software. Results are expressed as means ± standard error of the mean (SEM). N = number of culturing conditions and n = number of cells, if not stated otherwise. Sample sizes for experiments were selected to capture adequate technical variation (number of cells; numbers of fields of view; number of coverslips). All experiments were repeated at least three times.

## 3 Results

### 3.1 Effect of various substrates involved in energy metabolism on mitochondrial membrane potential

**Fig. 1:**
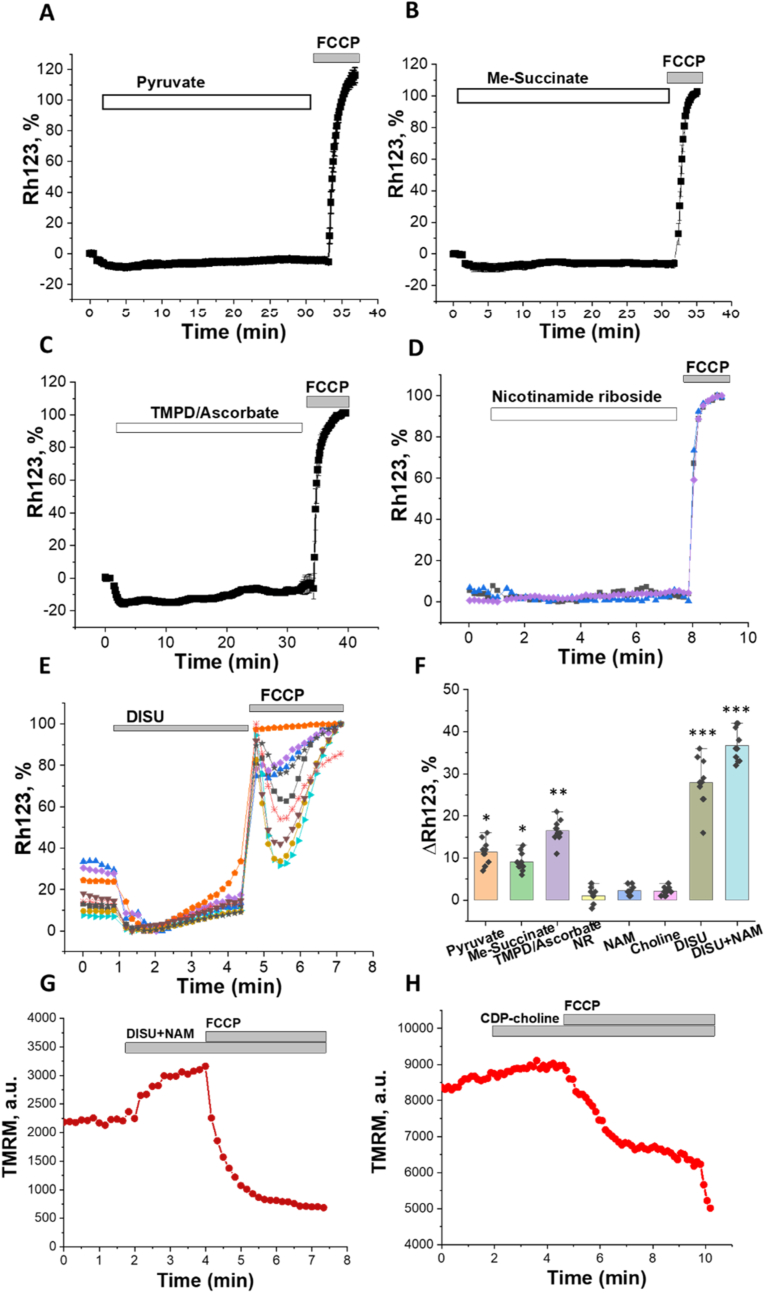
Effect of mitochondrial substrates and their combinations on mitochondrial membrane potential of primary neurons and astrocytes. Application of 5 mM pyruvate (A), 5 mM methyl succinate (B), 200 μM TMPD + 5 mM ascorbate (C), 40 μM NAM (D) or 50 μM DISU (E) induce decrease in Rh123 fluorescence which corresponds to mitochondrial hyperpolarization. d- 20 μM nicotinamide riboside had no effect on Rh123 fluorescence. F – Percentage of changes in Rh123 fluorescence in response to various compounds. G, H – application of 100 μM DISU + NAM or 100 μM CDP-choline change TMRM signal in mitochondria of primary neurons and astrocytes. 1 μM FCCP was added at the end of each experiment to induce mitochondrial depolarization for calibration of the signal. Data are represented as mean ± SEM. Unpaired (Mann-Whitney) t-tests, *p < 0.05, **p < 0.001, ***p < 0.0001.

In order to identify compounds which can activate energy metabolism, we used several substrates and activators to activate the TCA cycle and mitochondrial metabolism (succinate, pyruvate, Me-succinate, nicotinamide, choline chloride and combinations of these compounds). In the first stage of the experiments, we studied the effect of all compounds on the mitochondrial membrane potential (Δψm) of primary cortical neurons and astrocytes. Thus, in agreement with previously published data (Kovac et al., 2012, 2015; Abramov et al., 2004) application of 5 mM pyruvate or a membrane-permeable analogue of succinate - 5 mM methyl succinate - induced a 10–15 % increase of Δψm expressed as a decrease in Rh123 fluorescence (Fig. 1 A-B, F). Increase of Rh123 fluorescence in response to depolarization of mitochondria with 1 μM FCCP in the end of experiments confirmed the effect of pyruvate and succinate (Fig. 1 A, B). It should be noted that another substrate of the mitochondrial electron transport chain (ETC) 200 μM TMPD + 5 mM ascorbate also increased Δψm in primary neurons and astrocytes (Fig. 1 C). None of the forms of nicotinamide induced short-term changes in mitochondrial membrane potential (Fig. 1 D, F). Addition of choline chloride had only minor effects on Δψm (Fig. 1 F). However, a choline salt of succinic acid (2:1 - DISU) that improves succinate permeability induced a much more profound effect on Δψm (Fig. 1 E, F). Moreover, a combination DISU and nicotinamide (NAM), induced a further increase in Δψm in both neurons and astrocytes (Fig. 1 F).

Measurement of mitochondrial membrane potential with another fluorescent indicator - tetramethylrhodaminemethylester (TMRM) confirmed that even short-term application of 100 μM DISU+40 μM NAM induced an increase of Δψm in neurons and astrocytes (Fig. 1G and H). However, a derivative of choline – CDP-choline had almost no effect on the mitochondrial membrane potential. This confirms the importance of mitochondrial substrates in the maintenance of Δψm.

### 3.2 Changes in mitochondrial NADH pool and redox index

**Fig. 2:**
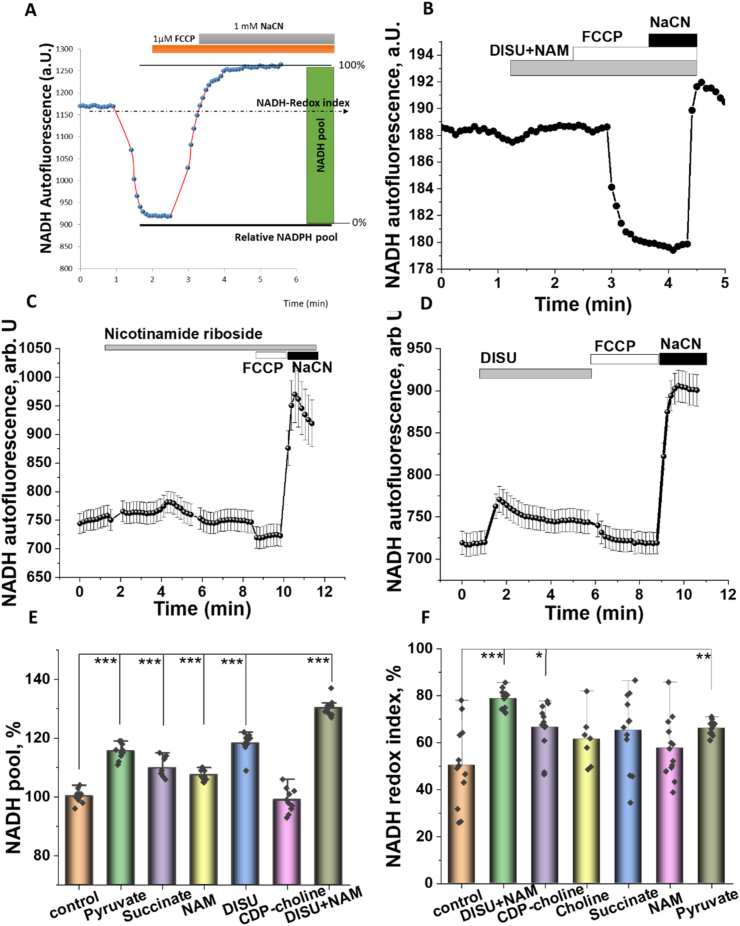
Mitochondrial substrates increase mitochondrial NADH level. A -Calculation of the mitochondrial NADH pool and NADH redox index after application of 1 μM FCCP and 1 mM NaCN. B, C- Application of 20 μM nicotinamide riboside or 50 μM DISU induced increase in NADH autofluorescence in primary co-culture neurons and astrocytes. Changes in mitochondrial NADH pool (D) or NADH redox index (E) of primary cortical neurons and astrocytes after 24 h incubation with 5 mM pyruvate, 5 mM succinate, 20 μM NAM, 50 μM DISU, 20 μM choline, 20 μM CDP-choline or 100 μM DISU + NAM. Data are represented as mean ± SEM. Unpaired (Mann-Whitney) t-tests, *p < 0.05, **p < 0.001, ***p < 0.0001.

Mitochondrial metabolism in live cells and in tissues can also be assessed by the measurement of mitochondrial redox index. To do this, we measured NADH autofluorescence signal in primary neurons and astrocytes. To separate mitochondrial NADH from NADPH and non-mitochondrial NADH, protonophore FCCP (to maximize the rate of respiration and NADH consumption, taken as 0%) and complex IV inhibitor NaCN (to block respiration and consumption of NADH, taken as 100%) were used (Fig. 2 A).

These measurements allowed us to assess two cellular characteristics which are connected to NADH: mitochondrial NADH content (pool) and redox index (balance between production and consumption of NADH in mitochondria (Barilani et al., 2022), (Fig. 2 A). Incubation of primary neurons and astrocytes with mitochondrial substrates (pyruvate, malate, succinate, or me-succinate) expectably changed NADH redox index (Fig. 2A–B) and increased mitochondrial NADH pool (Fig. 2 E, F) see also (Abramov et al., 2004). Interestingly, NAM, which can be used as precursor for NADH synthesis, had only moderate effect on the NADH pool (Fig. 2 C). DISU was much more effective than other substrates or NAM (Fig. 2 D). All compounds used induced an increase in NADH redox index with maximal effect observed for DISU + NAM (Fig. 2A–D,E). Thus, mitochondrial substrates and their combinations in the form of DISU + NAM induced an increase in NADH levels and NADH redox index suggesting activation of energy metabolism.

### 3.3 Supplementation of neurons and astrocytes with various substrates for energy metabolism increased ATP

**Fig. 3:**
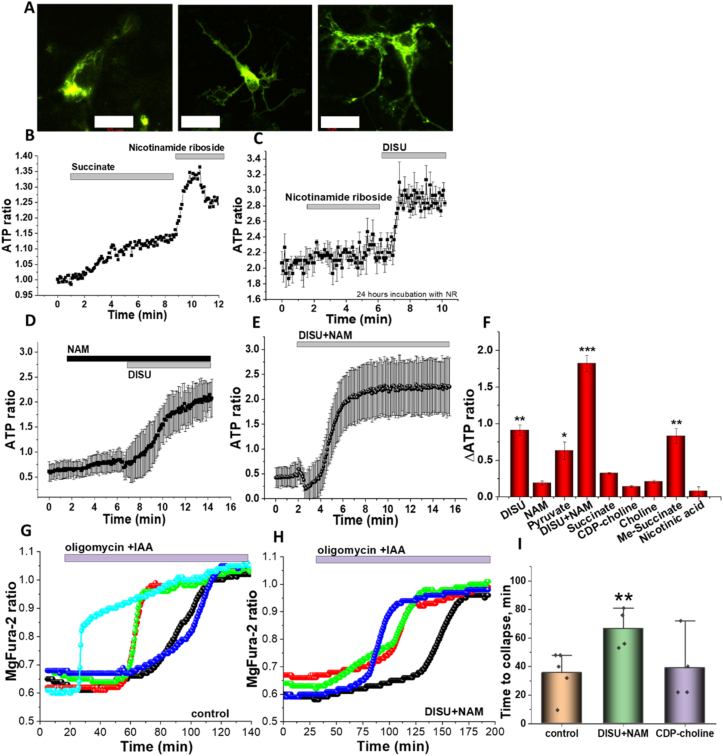
Mitochondrial substrates increase ATP level in primary neurons and astrocytes. A – images of AT1.03 probe distribution in primary neurons and astrocytes. Scale bar 20 μM. Application of 5 mM succinate +20 μM nicotinamide riboside (B) or 20 μM nicotinamide riboside + 50 μM DISU (C), 20 μM NAM + 50 μM DISU or 100 μM DISU + NAM, D) increase AT1.03 ratio in primary neurons and astrocytes. E − effect of incubation of the various compounds on the ATP level (presented as increase in AT1.03 ratio) in primary neurons and astrocytes. Changes in the magFura-2 ratio of primary neurons and astrocytes after application of 2 μg/ml oligomycin +20 μM IAA in control (F) or after incubation of cells with 100 μM DISU + NAM (G). H – Time to cell collapse from the application of 2 μg/ml oligomycin +20 μM IAA in control neurons and astrocytes or cells pre-treated with 100 μM DISU + NAM or 100 μM CDP-choline. Data are represented as mean ± SEM. Unpaired (Mann-Whitney) t-tests, *p < 0.05, **p < 0.001, ***p < 0.0001.

Using a mitochondrially targeted version of the genetically-encoded ATP-sensing probe AT1.03 in primary neurons and astrocytes (Fig. 3 A) we have found that application of sodium succinate alone (1 mM; N = 4 experiments) induced only small 1.2-fold increase in [ATP]c of neurons and astrocytes (Fig. 3 A, B). [ATP]c of neurons and astrocytes (Using a mitochondrially targeted version of the genetically-encoded ATP-sensing probe AT1.03 in primary neurons and astrocytes (Fig. 3 A) we have found that application of sodium succinate alone (1 mM; N = 4 experiments) induced only small 1.2-fold increase in [ATP]c of neurons and astrocytes (Fig. 3 A, B). Interestingly, subsequent addition of nicotinamide riboside induced a more profound increase in ATP levels (Fig. 3 B). Application of nicotinamide riboside or nicotinamide (NAM) alone induced only moderate rise in [ATP]c (Fig. 3 C, D); however, these applications augmented the effect of DISU. Importantly, addition of the same concentration (20 μM) nicotinic acid did not induce any significant rise in [ATP]c of neurons and astrocytes (Fig. 3 D, F). Choline bitartrate or CDP-choline also induced significant but moderate rise in ATP levels (0.21 ± 0.02 and 0.14 ± 0.012 accordingly; N = 4 experiments; Fig. 3 F). The presence of DISU + NAM induced an 8-fold enhancement compared to the effect of sodium succinate on [ATP]c in neurons and astrocytes (Fig. 3 E, F) that suggests that combination of substrates is more effective for ATP synthesis than each of the substrates alone.

### 3.4 DISU + NAM increased energy capacity of primary neurons and astrocytes

ATP is stored as a magnesium complex in the cells. Upon hydrolysis of ATP, Mg2+ is released from the Mg2+-ATP complex; therefore, measurement of the changes in the cellular free magnesium using the Mg2+ sensitive fluorescent probe MagFura-2, can be used as an indirect reporter for the rate of ATP consumption (Leyssens et al., 1996). Application of inhibitors of glycolysis and/or oxidative phosphorylation blocks ATP production in cells, which leads to utilization of the available ATP in the cell, and a subsequent Mg2+ release. In addition to binding to Mg2+, the MagFura-2 dye is also a low-affinity Ca2+ indicator that can help detect high cytosolic calcium rise in the time of cell lysis, i.e. the energetic collapse due to a total cellular ATP depletion and the inability of the cell to maintain Ca2+ homeostasis This enables the estimation of the cellular energy capacity. In our experiments, incubation of the cells with DISU + NAM (30 min) increased the time to neuronal or astrocytic collapse (Fig. 3 g, h, i), strongly suggesting the increase of energy capacity of these cells compared to untreated cells. It should be noted that incubation of the cells with citicoline (30 min) did not change the time from inhibition of ATP synthesis to cell collapse, suggesting only minor effects of this compound on the maintenance of energy capacity of the cells (Fig. 3 i). Thus, providing the cells with combination of DISU and NAM for energy metabolism increased ATP levels and enhanced the energy capacity of primary neurons and astrocytes.

### 3.5 Energy supplementation protects neurons against glutamate-induced excitotoxicity

Glutamate is one of the major neurotransmitters in the brain. A large number of conditions, which lead to the exposure of neurons to excessive concentration of glutamate, induce excitotoxic cell death (Choi, 1992). Glutamate excitotoxicity induces loss of mitochondrial membrane potential (Δψm) and energetic collapse followed by cell death (Abramov et al., 2008, 2010).

**Fig. 4:**
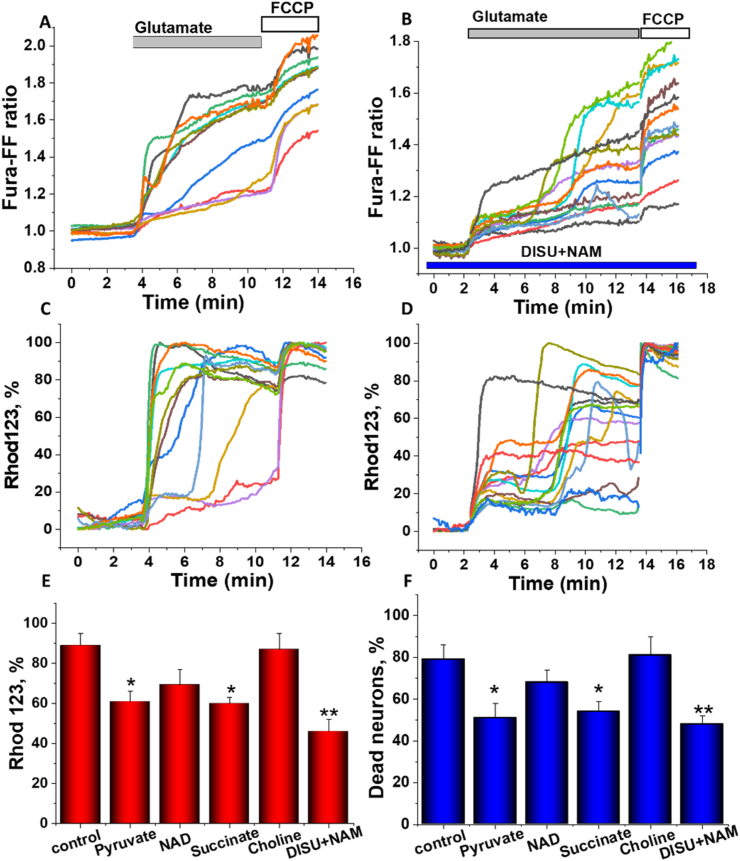
Effect of compounds augmenting energy metabolism on glutamate-induced excitotoxicity. A-D, Simultaneous measurements of changes in [Ca2+]c (Fura-FF ratio) and Δψm (relative Rh123 fluorescence, in response to 50 μM glutamate recorded in single neurons. An increase in Rh123 fluorescence reflects mitochondrial depolarization. The histogram in E represents changes in the mean Rh123 fluorescence in neurons after 10 min of exposure to 50 μM glutamate. F- Percentage of dead (PI positive) neurons 24 h after application of 50 μM glutamate. E-f comparative responses to pyruvate (5 mM), succinate (5 mM), NAM (300 μM), choline or DISU + NAM (100 μM) added to cells 10 min before glutamate application. Data are represented as mean ± SEM. Unpaired (Mann-Whitney) t-tests, *p < 0.05, **p < 0.001, ***p < 0.0001.

Application of 50 μM glutamate to mature (≥12 days in vitro) hippocampal neurons causes a response that consists of an initial transient rise in [Ca2+]c followed after a several minutes by a secondary delayed increase to a plateau (Fig. 4 A; n = 98 neurons) (Abramov et al., 2008; Stout et al., 1999). Simultaneous measurements of mitochondrial potential showed that the delayed secondary increase in [Ca2+]c is accompanied by a progressive and possibly complete loss of mitochondrial potential in Fig. 4 C (Abramov et al., 2008; Vergun et al., 1999). In agreement with previously published data on the effect of succinate on the excitotoxicity (Abramov et al., 2008), pre-incubation of the cells with pyruvate, succinate or DISU + NAM did not change the glutamate-induced calcium signal but significantly reduced mitochondrial depolarization (for DISU + NAM from 89 ± 6% to 57.5 ± 5.1%; n < 0.001; n = 107 neurons; Fig. 4 C, D). The effect of high doses of glutamate on mitochondrial membrane potential is mediated by the activation of DNA repairing enzyme PARP which consumes NAD and restricts production of NADH for mitochondrial respiration (Abramov et al., 2008; Duan et al., 2007). Pre-incubation (30 min, 300 μM) of the cells with NAD induced only a small reduction of the effects of 50 μM glutamate on Δψm (to 63.4 ± 7.4%; n = 89; p < 0.05; Fig. 4 C). Thus, application of compounds that induce ATP increase protect neurons against glutamate-induced Δψm collapse.

Importantly, pyruvate, succinate and, even more effectively, DISU + NAM, protected neurons against glutamate-induced cell death (Fig. 4 F). Separately, NAM or choline, which were not highly effective in increasing ATP in neurons and astrocytes alone, also did not show any effect on glutamate-induced neurotoxicity (Fig. 4 F).

### 3.6 DISU + NAM-induced ATP increase protects neurons and fibroblasts with familial forms of Parkinson's disease against cell death

**Fig. 5:**
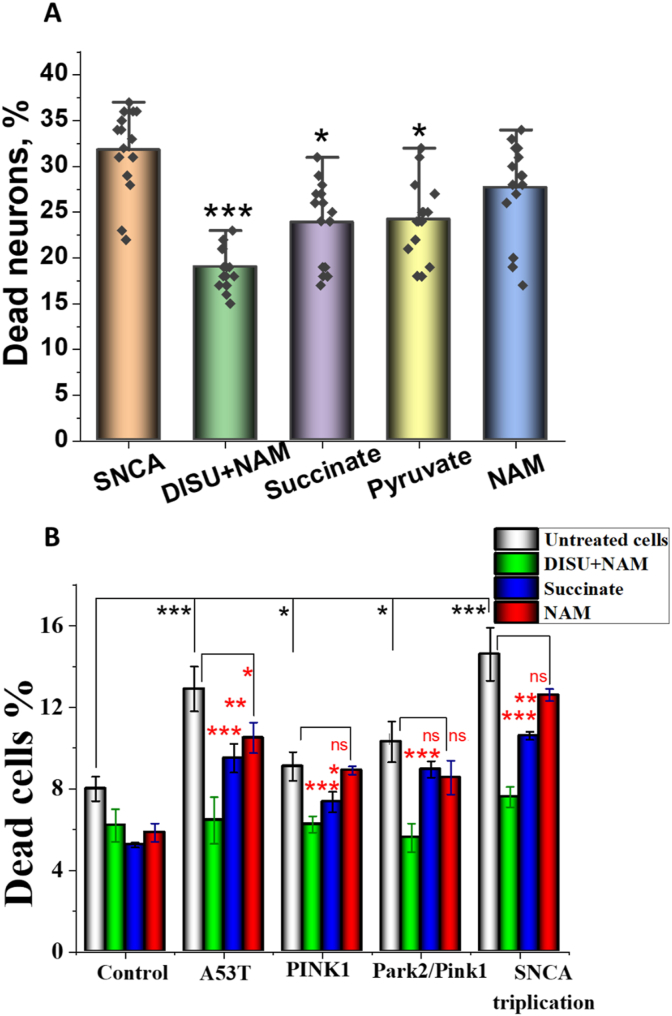
Mitochondrial substrates protect neurons and fibroblasts with familial forms of Parkinson's disease against cell death. (A) A 72-h incubation of iPSC-derived neurons with *snca* triplication with 100 μM DISU + NAM, 5 mM pyruvate, 5 mM succinate or 20 μM NAM reduced the number of dead neurons. (B) Effect of 72-h incubation with 100 μM (DISU + NAM), 5 mM succinate or 20 μM NAM on the viability of patients' fibroblasts with familial forms of Parkinson's disease. Data are represented as mean ± SEM. Unpaired Mann-Whitney t-tests (A) and one-way ANOVA with Bonferroni post hoc test (B), Increase (black *), decrease (red *). *p < 0.05, **p < 0.001, ***p < 0.0001. (For interpretation of the references to colour in this figure legend, the reader is referred to the Web version of this article.)

Human iPSC-derived neurons with familial forms of Parkinson's disease (snca triplication), and healthy controls were treated with succinate, pyruvate, NAM, or DISU + NAM for 72 h. Cell death (necrosis) was measured using Hoechst (total number of cells) and PI (dead cells) labelling. Percentage of dead human neurons at basal was compared with the levels of cell death in treated cells. We have found that supplementation of the human iPSC-derived neurons with snca triplication mutation with DISU + NAM, but not with other compounds, significantly reduced the percentage of dead neurons (Fig. 5 A).

To confirm these effects, we used patient's fibroblasts with various familial forms of Parkinson's disease (Komilova et al., 2022). Incubation of the cells with DISU + NAM, pyruvate or succinate for 72 h significantly protected human fibroblasts with PINK1, PINK1/PARK2, A53T and SNCA mutations against cell death (Fig. 5 B). It should be noted that in all fibroblasts the most effective was the DISU + NAM combination.

## 4 Discussion

Here we show that provision of mitochondrial substrates such as pyruvate or succinate could not only change mitochondrial membrane potential or redox index of neurons and astrocytes but could also significantly increase the ATP levels in these cells. Importantly, a specific combination of the three substrates, as found in DISU + NAM, leads to an even more pronounced effect on ATP, compared to the effect of the separate components. Interestingly, succinate itself is non-permeable to the cell membrane and to increase its membrane permeability to ultimately increase bioavailability, a salt has been synthesized to form DISU, a choline succinate salt (2:1). The salt form of choline, choline chloride was put in a reaction with succinic acid to form the choline succinate salt (2:1). Previously, a study has shown a protective effect of succinic acid salt of choline in the brain of a rat, an animal model of total ischemia (Pomytkin and Semenova, 2005). In ischemic conditions, a major event of the damaging cascade is due to so called glutamate excitotoxicity, when too much glutamate has been released and this leads to overstimulation of neurons and neuronal death. In Parkinson's disease, and many other neurodegenerative disorders, the major for glutamate-induced neurotoxicity is the impaired glutamate reuptake of the glial cells (Iovino, 2020).

Glutamate excitotoxicity is an energy demanding process due to a massive activation of the ionic ATPases and disruption of the process of energy production (Abramov et al., 2010; Nicholls et al., 2007). Importantly, in our experiments energy substrate supplementation did not dramatically change the glutamate-induced calcium signal, but it did reduce its effect on Δψm that can be sufficient to protect the cells from mitochondrial energy collapse. Provision of ATP to the neurons and elongation of the period to complete energy deprivation and consequent cell collapse is the most likely mechanism of neuronal protection.

The mechanism of pathology of Parkinson's disease is very complex and includes multiple cellular and molecular players, e.g. oxidative stress, impaired calcium signaling, mitochondrial dysfunction (Angelova et al., 2017). Currently it is not clear which of the triggers is the most important for the initiation and the development of the disease. However, mitochondrial dysfunction and alteration of energy metabolism in Parkinson's disease was shown not only for neurons but also for fibroblasts and even for myocytes (Abramov et al., 2011; Yao et al., 2011). Here we found that energy supplementation is protective not only for neurons but also for patients' fibroblasts with familial forms of Parkinson's disease.

Importantly, it should be noted that in brain cells the metabolism of glucose is used for 1) energy production in the form of ATP through the processes of glycolysis and oxidative phosphorylation and 2) in the pentose phosphate pathway for NADPH production which in turn is mostly used for cellular redox status maintenance (Esteras et al., 2023). Considering this, changes in the energy metabolism can also affect the neuronal redox balance. Thus, supplementation of cells with energy production substrates may have indirect stabilizing effect on the redox balance and cell protection can be partially mediated by this mechanism as well.

## Informed consent statement

We have ethical approval for investigating patients with informed consent and taking skin samples for research approved by University College London Hospital ethics committee (Number: 07/N018).

## Funding

A.Y.V. was supported by the grant of Russian Science Foundation (22-15-00317).

## CRediT authorship contribution statement

**Andrey Y. Vinokurov:** Investigation, Validation, Formal analysis. **Marina Y. Pogonyalova:** Investigation, Formal analysis. **Larisa Andreeva:** Conceptualization, Resources. **Andrey Y. Abramov:** Conceptualization, Methodology, Investigation, Validation, Formal analysis, Visualization, Supervision, Writing – original draft, Writing – review & editing. **Plamena R. Angelova:** Conceptualization, Methodology, Investigation, Validation, Formal analysis, Visualization, Supervision, Writing – original draft, Writing – review & editing, All the authors read and approved the final version of the manuscript.

## Declaration of competing interest

L.A. is an employee of Mitocholine Ltd. All other authors declare no conflict of interest. The funder had no role in the study's design, the collection, analysis, or interpretation of data, the writing of the manuscript, or the decision to publish the results.

## Data Availability

No data was used for the research described in the article.
